# SHI: a framework for spatial harmonic imaging

**DOI:** 10.1038/s41598-026-37029-5

**Published:** 2026-01-29

**Authors:** Jorge Luis Beltran Diaz, Jan G. Korvink, Danays Kunka

**Affiliations:** Institute of Microstructure Technology (IMT), Karlsruhe Institute of Technology (KIT), Hermann-von-Helmholtz-Platz 1, 76344 Eggenstein-Leopoldshafen, Baden-Württemberg Germany

**Keywords:** Spatial harmonic imaging, Software development, Multicontrast, X-ray, Engineering, Mathematics and computing, Optics and photonics

## Abstract

Despite the growing interest in multicontrast X-ray imaging, *spatial harmonic imaging* remains limited by a lack of specialized computational resources. In this paper, we present *SHI*, a high-performance software framework that covers the range from data acquisition to processing in spatial harmonic imaging experiments. *SHI* is an open-source software package that facilitates the acquisition of precise measurement data and streamlines the workflow, ensuring that data can be efficiently organized, processed, and visualized, leading to high quality results. In addition, *SHI* includes higher-order harmonic extraction. Preliminary results show that spatial harmonic imaging improves experimental robustness and retrieves refraction and scattering information, albeit with reduced resolution. However, using these lower-resolution images enables faster CT reconstruction with fewer projections, while preserving essential sample features and allowing a substantial reduction in exposure levels. This research focuses on the acquisition methodology and the subsequent data processing for contrast retrieval and multicontrast computed tomography.

## Introduction

**Fig. 1:**
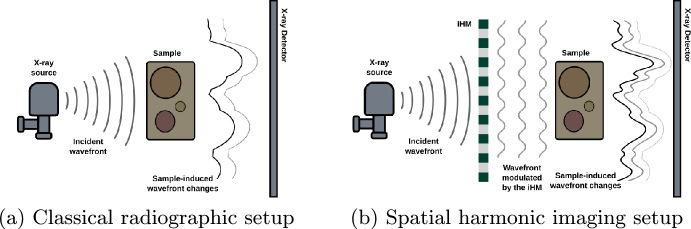
Experimental setups illustrating two radiographic configurations. (**a**) Conventional radiographic setup without optical component. (**b**) Modified radiographic setup incorporating a periodic grating to modulate the wavefront emitted by the X-ray source (spatial harmonic imaging setup).

Experimental multicontrast X-ray imaging has been widely developed; examples are propagation-based X-ray imaging^1^, grating-based X-ray interferometry^2^, edge-illumination X-ray phase contrast imaging^3^, speckle-based X-ray imaging, Talbot-Lau X-ray interferometry^4^, diffraction-enhanced X-ray imaging^5^, and mesh-based X-ray imaging^6^. The experimental configuration comprises a conventional radiography setup which can be divided into three distinct parts: the X-ray source, the imaging system, and the detector [Fig. 1a ]. The primary distinction between these techniques lies in the modifications applied to the imaging systems. These modifications may involve one or more fixed or movable optical components, the application of mathematical data processing algorithms, or a combination of both, all of which are aimed at enhancing the quality of the final images.

The well-implemented modalities mentioned above have been accompanied by both hardware and computational tools to increase their performance and precision^7–14^.

Spatial harmonic imaging is a multicontrast X-ray imaging method that retrieves information from absorption, scattering, and differential phase contrast using a periodic optical component (hereafter referred to as a periodic modulator or modulator) and Fourier transform techniques as imaging system^15–17^. It employs Fast Fourier Transform routines to obtain a set of harmonics, that is, a set of equidistant maxima in the spatial-frequency domain (Fourier space) corresponding to the periodic modulation of the optical component and assumed to be band-limited. These harmonics must then be extracted and separated to obtain different contrast mechanisms. Several variations of the method have been developed in combination with other techniques or mathematical manipulations to increase the resolution of images and gain sensitivity^6,18–21^. Despite continuous improvements, spatial harmonic imaging lacks a unified computational framework that covers the entire measurement pipeline from data acquisition to comprehensive data analysis. This method has significant potential for widespread applications. However, its adoption has been constrained by previously fragmented manual data collection and analysis processes. The uniformity and practical success of this method, particularly in improving reproducibility and encouraging a wider adoption of spatial harmonic imaging across research environments, depend on the availability of a computational tool capable of addressing this critical gap.

To overcome this bottleneck, we introduce *SHI*, a robust, open-source and user-friendly software package developed for spatial harmonic imaging using Hartmann masks^22–24^, which can be easily extended to other periodic modulators. SHI automates and integrates the complete workflow, allowing efficient, straightforward and high-performance contrast retrieval not only for multicontrast images, but also for multicontrast computed tomography applications^25,26^. Here, we detail the architecture, functionality, and validation of the SHI, demonstrating its use with hazelnuts as a representative sample to illustrate internal structural heterogeneity.

## Results

### Experimental setup

In this experimental setup, which closely resembles a conventional radiographic system (Fig. 1a), a periodic optical component periodic modulator) is placed within the incident X-ray beam. The component modulates the wavefront to create discrete beamlets that irradiate the sample, and the detector records the sample-induced distortions of these beamlets (Fig. 1b ).

The X-ray source for validating our framework is the microfocus Excillum MetalJet D2+. The Excillum MetalJet X-ray sources represent an advanced technology in X-ray generation. Unlike traditional X-ray sources that rely on solid anodes, the MetalJet system employs a continuously renewed liquid metal jet as its anode. This design not only enhances heat dissipation but also allows the source to operate at higher power levels, resulting in increased brightness and improved flux. Consequently, these sources are particularly well suited for high-resolution imaging applications, such as spatial harmonic imaging, where a stable and high-quality X-ray beam is essential for optimal data acquisition and processing.

The periodic modulator, an inverted Hartmann mask, comprises a regular array of absorbing metal pillars arranged on a low-absorption substrate. In this configuration, the pillars obstruct radiation, permitting passage only through the interstitial spaces, thus generating a mesh-like intensity pattern. In the resultant image, the areas corresponding to the pillars exhibit low intensity, whereas the regions between them display high intensity. This approach maximizes photon collection, as the majority of the beam impinges on the unobstructed regions, thereby increasing the flux efficiency and improving the signal-to-noise ratio (SNR) in the final images without compromising the spatial or angular resolution.

The fabrication of the inverted mask is carried out through a multistep process: first, a layer of photoresist is deposited on a substrate (in this case, Kapton sputtered with Au as a seed layer), then it is exposed to UV radiation to define a solid polymer pattern, and finally, this pattern is used to form gold pillars via electroplating. Typical parameters include a pillar height of more than $40\,\upmu m$ (suitable for the energy ranges mentioned in the setup) and a period of $50\,\upmu m$, adjusted based on the detector’s pixel size ($49.5\,\upmu m$)^23,27^.

For our experimental investigations, a hazelnut was selected as the test sample due to its availability and moderately complex internal structure.

### Image acquisition

**Fig. 2:**
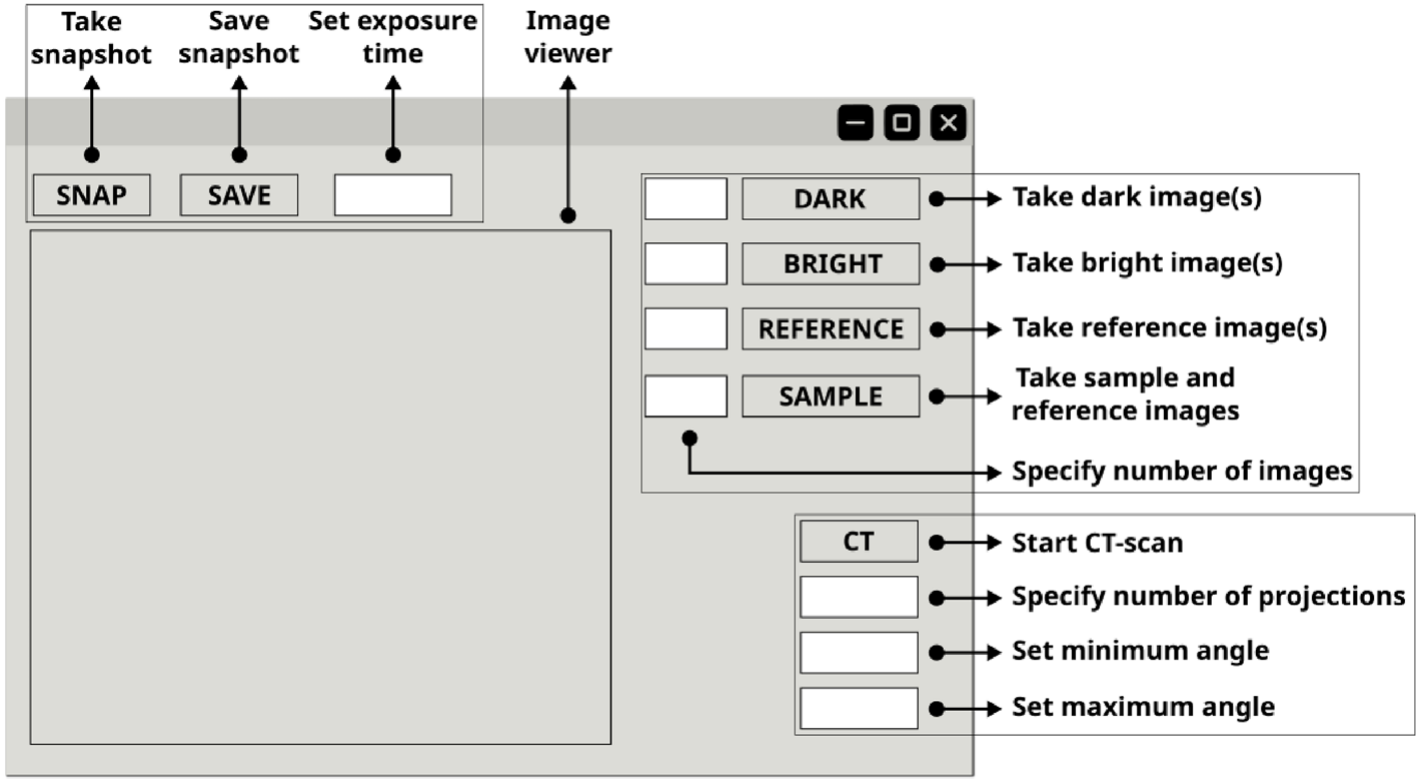
Graphical user interface (GUI) designed for the image acquisition procedure. The GUI is straightforward and user-friendly, featuring two primary operation modes: single projection imaging and computed tomography (CT). Acquisition of dark, bright, and reference images is common to both modes. Additionally, the interface includes real-time visualization of acquired images, camera controls for adjusting exposure time, and snapshot functionality, allowing temporary image inspection without affecting the organized measurement directories.

From an experimental perspective, proper execution of this method requires the acquisition of the following images:

1. Dark image: image with the X-ray source OFF (Fig. 12a)
2. Bright image: image with the X-ray source ON (Fig. 12b)
3. Reference image: image of the modulator (Fig. 12c)
4. Sample image: image of the sample and modulator (Fig. 12d).

For the acquisition process, we developed a graphical user interface (GUI) based on an *event-driven architecture* that simulates a camera (see Fig. 2). In this design, initiating the acquisition of each image is as simple as pressing a corresponding button (e.g., the dark button for dark-field images). Although the sequence of measurements may vary according to the user’s preferences, to minimize errors and ensure optimal experimental results, it is crucial that if the sample image is acquired first, the modulator remains fixed when the sample is removed; conversely, if the reference image is acquired first, the modulator must remain stationary when the sample is introduced. Additionally, to enhance the SNR, it is advisable to capture multiple images per measurement step and average them during postprocessing. The images acquired for a hazelnut as a test object are shown in Fig. 12.

### Image processing and contrast retrieval

After the measurements have been acquired, the final procedure is data processing. To ensure successful processing, the following steps must be implemented:

**Fig. 3:**
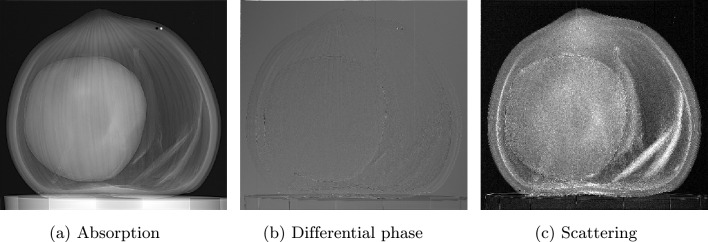
Spatial harmonic images showing three different contrast modalities: (**a**) absorption contrast, (**b**) differential phase contrast, and (**c**) scattering contrast.

1. Dark-field and bright-field corrections to reference and sample images
2. Fourier transform to flat-field-corrected reference and the sample images
3. Extraction, separation and labeling of each harmonic of both Fourier-transformed reference and sample images
4. Inverse Fourier transform of each harmonic
5. Reference correction consisting to subtract the corresponding harmonic contribution of the reference image from the sample image.
6. In case of several measurements, calculate the average and export
7. In the case of computed tomography, each reference-corrected harmonic should be saved separately in dedicated directories to facilitate subsequent reconstruction.

Fourier analysis plays a crucial role in spatial harmonic imaging data processing by decomposing the acquired signals into their spatial harmonic components, thereby extracting key contrast information from the images. Although the application of Fourier analysis to an individual image does not present significant challenges, it is essential to compare the harmonic content of the sample containing the optical component with that of the optical component alone. This comparative analysis is performed after recovering each harmonic via the inverse Fourier transform. Three distinct approaches are available for this process:

1. Manual processing: this is the current spatial harmonic imaging method, which can be time-consuming even for a single sample. For instance, processing 10 samples would further increase the time required, which is a critical concern in clinical and dynamic process investigations.
2. Semi-automatic processing: in this mode, initial parameters are defined manually, after which calculations are automated. Although this approach reduces overall processing time, the parameter selection may vary between samples, leading to potential ambiguities.
3. Fully automatic processing: this approach minimizes the processing time to mere seconds, depending on various factors, and enables real-time visualization. It also avoids the ambiguities introduced by the selection of variables. A key advantage is that it abstracts the user from the underlying computational details, allowing researchers to conduct experiments without engaging in coding. Currently, there is no open-source solution that implements this level of automation in the literature.

**Fig. 4:**
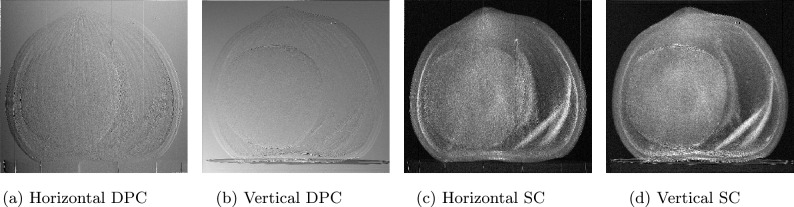
Preferential directions of (**a,b**) differential phase contrast (DPC) and (**c,d**) scattering contrast (SC). Images (**a,c**) correspond to the horizontal direction, derived from the $(\pm 1,\,0)$ harmonics, and reflect changes along this direction. Images (**b,d**) represent the vertical direction, derived from the $(0,\,\pm 1)$ harmonics, and reflect variations along this direction.

We adopted fully automatic processing to retrieve the multicontrast images. In order to carry out every step efficiently, a command-line interface package was developed for data analysis, which follows a *Pipes-and-Filters* architecture to keep high modularity and flexible integration with further developments. This package can be used in other laboratories, as long as raw data directories are organized in the same way as obtained in the acquisition procedure.

The contrasts obtained using the spatial harmonic imaging technique include absorption contrast, scattering contrast, and differential phase contrast^15–17^. These three contrasts are specifically retrieved through inverse Fourier transforms of the harmonic orders $(m=0,\,n=0)$, $(m=0,\,n=\pm 1)$, and $(m=\pm 1,\,n=0)$, respectively [see “Equations”), after applying reference correction. Each pair of harmonic indices (m,n) denotes the spatial-frequency order of the Fourier components generated by the periodic optical component modulation pattern. It should be noted that the fundamental and first-order harmonics are not the only extractable ones; second-order harmonics, namely those of order $(m=\pm 1,\,n=\pm 1)$, are also accessible. Our software is automatically capable of extracting and organizing these harmonics according to their intended use, whether for two-dimensional projections or computed tomography reconstruction.

Absorption contrast images are shown in Fig. 3a for the hazelnut sample. Regarding scattering and differential phase contrasts, we have included only bidirectional cases (Fig. 3c , Fig. 3b ). Since the optical component has a two-dimensional periodic structure, bidirectional scattering and differential phase contrasts are obtained by averaging vertical and horizontal directions, extracted from harmonics $(m=0,\,n=\pm 1)$ and $(m=\pm 1,\,n=0)$, respectively. These separate directional contrasts are shown in Fig. 4.

**Fig. 5:**
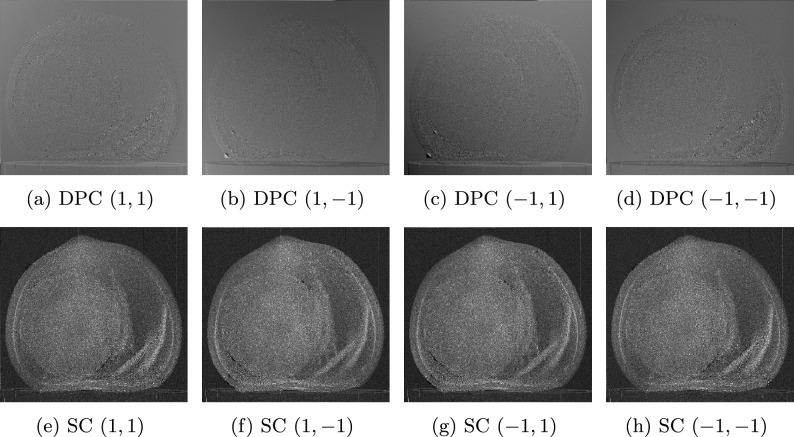
Diagonal directions corresponding to the second-order harmonics $(\pm 1,\,\pm 1)$, representing second-order contributions to (a–d) differential phase contrast (DPC) and (e–h) scattering contrast (SC).

The diagonal directions, corresponding to second-order harmonics $(\pm 1,\,\pm 1)$, contain second-order contributions to scattering and differential phase contrasts^28,29^. These second-order scattering and differential phase contrasts are shown in Fig. 5.

### Computed tomography for spatial harmonic imaging

It is often of interest to obtain a three-dimensional perspective of the sample under investigation, and in X-ray imaging, computed tomography (CT) is commonly used for this purpose. Classical CT reconstruction algorithms were originally developed for the configuration shown in Fig. 1a under different geometries, such as parallel, fan, and cone beams. However, when an optical component, such as the inverted Hartmann mask used in our setup (Fig. 1b ), is introduced either before or after the sample, certain aspects of the CT geometry assumptions must be reconsidered (see “Computed tomography reconstruction” for a detailed discussion of the CT geometry considerations introduced by the optical component). Specifically, in our case, where a cone-beam geometry is used, the presence of the optical component directly affects the definition of the effective pixel size of the detector.

**Fig. 6:**
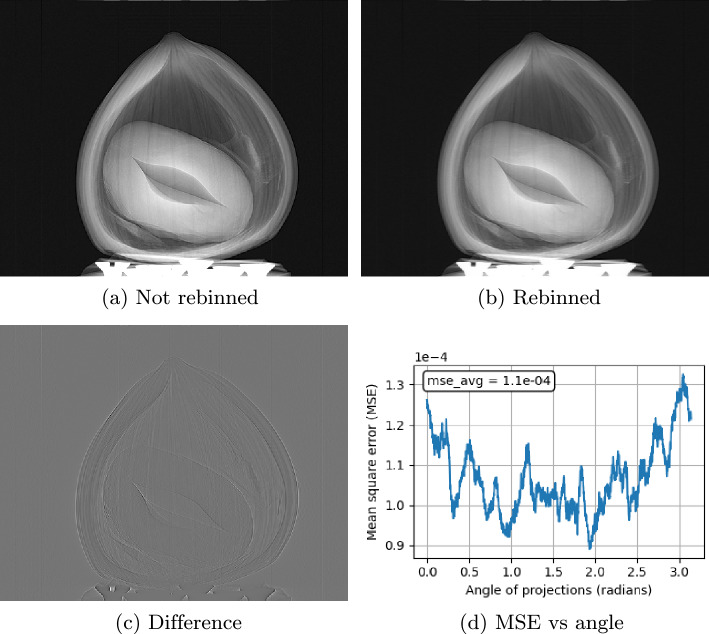
Example of a projection at angle $0^{\circ }$. (**a**) Non-rebinned projection, (**b**) rebinned projection for parallel-beam reconstruction, (**c**) difference between non-rebinned and rebinned projections, (**d**) mean squared error (MSE) as a function of projection angle.

Following the scanning procedure, the next step is to reconstruct the computed tomography. In cone-beam geometries, depending on the specific experimental conditions, it is sometimes possible to use algorithms originally designed for other geometries, such as parallel beams.

When employing a parallel beam reconstruction algorithm on cone beam data, the acquired projections must first be rebinned using a coordinate transformation^30^. Figure 6 illustrates both the non-rebinned (Fig. 6a ) and rebinned (Fig. 6b ) data, Fig. 6c shows their difference, and Fig. 6d reports the mean squared error (MSE) for each projection angle. The overall average MSE is minimal (Fig. 6d ), indicating that even without an appropriate rebinning routine, fast parallel beam algorithms such as *Gridrec-MS*^31^ produce final reconstructions that are accurate and reliable.

**Fig. 7:**
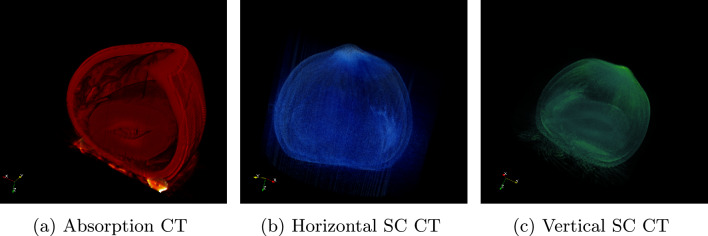
Computed tomography of hazelnut, showing (**a**) absorption contrast, (**b**) horizontal scattering contrast (SC), and (**c**) vertical scattering contrast (SC). Reconstructions were performed separately for horizontal and vertical orientations.

In Fig.  7, we have reconstructed a hazelnut tomography for the absorption and directional scattering contrasts, using the *Gridrec-MS* algorithm with a *Ram-Lak* filter. It is well established that the number of projections acquired at distinct angles fundamentally determines the final reconstruction quality, and specific sampling criteria dictate the minimum required number of projections. Equally important is the use of iterative reconstruction algorithms that can substantially enhance image fidelity. However, the deployment of such algorithms often requires significant computational resources that may not be readily available.

In the case of spatial harmonic imaging, the reduction in dimensionality of the final images (through harmonic-based filtering) helps to significantly decrease the number of projections required.

**Fig. 8:**
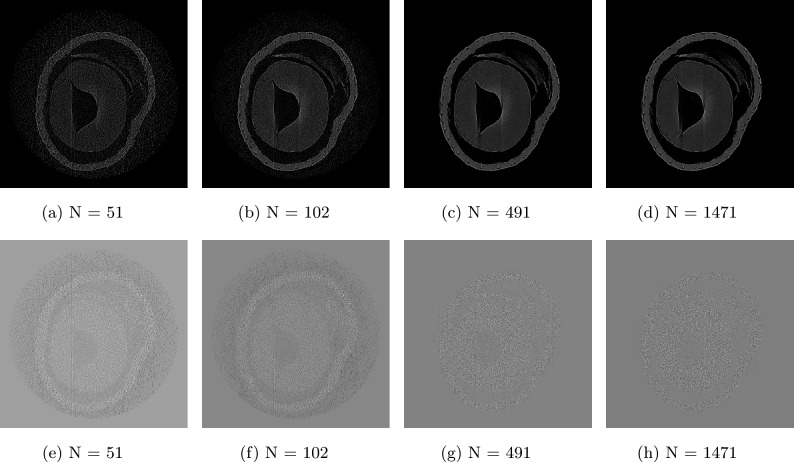
Evaluation of computed tomography (CT) reconstruction quality at slice 262. (**a–d**) Reconstructions performed using N = 51, 102, 491, and 1471 projections, respectively. (**e–h**) Corresponding difference images compared to the reference reconstruction using 2942 projections.

**Fig. 9:**
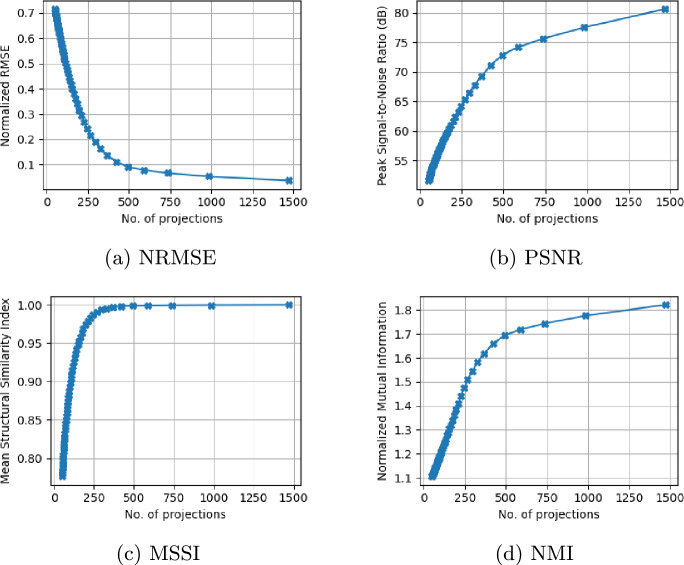
Statistical metrics evaluating the quality of CT reconstructions as a function of the number of projections. Metrics include (**a**) normalized root mean square error (NRMSE), (**b**) peak signal-to-noise ratio (PSNR), (**c**) mean structural similarity index (MSSIM), and (**d**) normalized mutual information (NMI), computed relative to the reference reconstruction using 2942 projections.

This effect is illustrated in Fig.  8a –8d , which present an intermediate slice of tomographic reconstructions performed with different numbers of projections. Using a 2942 projection reconstruction as a reference, Fig.  8e –8h show the difference images between the reference and each of the lower number of projection reconstructions. Information loss for reconstructions with the number of projections $N>490$ is effectively negligible and does not result in any perceptible loss of structural detail. Figure 9 summarizes the quantitative metrics, including normalized root mean square (NRMSE) (Fig. 9a ), peak signal-to-noise ratio (PSNR) (Fig. 9b ), mean structural similarity index (MSSIM) (Fig. 9c ) and normalized mutual information (NMI) (Fig. 9d )—computed across all slices relative to the 2942 projection baseline. The normalized RMSE decreases rapidly with increasing projection count, whereas the PSNR, MSSIM, and NMI all increase sharply beyond a certain number of projections.

Furthermore, the magnification effects inherent to the cone-beam geometry are naturally mitigated by the projected period of the optical component. This phenomenon is strongly dependent on the projected period: for a given projected period, features smaller than a certain size become unresolved, as variations induced by magnification become negligible once the resolution is reduced to the limits defined by each harmonic. The mitigation of magnification effects in SHI arises naturally from the sampling imposed by the projected period $d_p$ of the optical component. Because each harmonic is reconstructed within a band-limited region of spatial frequencies defined by $1/d_p$, geometric variations smaller than this scale—such as those introduced by cone-beam magnification—become negligible after harmonic filtering. For instance, two identical spherical features located at different positions along the beam propagation direction would appear with different apparent sizes in a standard cone-beam projection. However, after Fourier sampling and harmonic isolation, both features appear with the same apparent size, since magnification-induced differences fall below the effective resolution defined by $d_p$. This effect depends strongly on the position of the optical component, which sets the projected period and thus the scale at which magnification is suppressed. Consequently, the system can be treated as effectively parallel-beam for reconstruction purposes, as residual divergence effects remain below the harmonic sampling limit.

## Methods

To achieve satisfactory results using the spatial harmonic imaging technique can be challenging and time-consuming due to the extensive measurement processing involved. As mentioned above, it is imperative to acquire at least four different measurements: a dark-field image, a bright-field image, a reference image, and a sample image.

Ensuring an organized and efficient acquisition of experimental data is crucial for executing subsequent data processing without inconsistencies or errors. Data processing includes specifying regions of interest (ROI), performing flat-field corrections, applying Fourier routines, extracting harmonics, and finally executing a reference correction such that the harmonics extracted from the images containing the sample and the optical component are accurately adjusted using those reference images (see “Framework for contrast retrieval”).

**Fig. 10:**
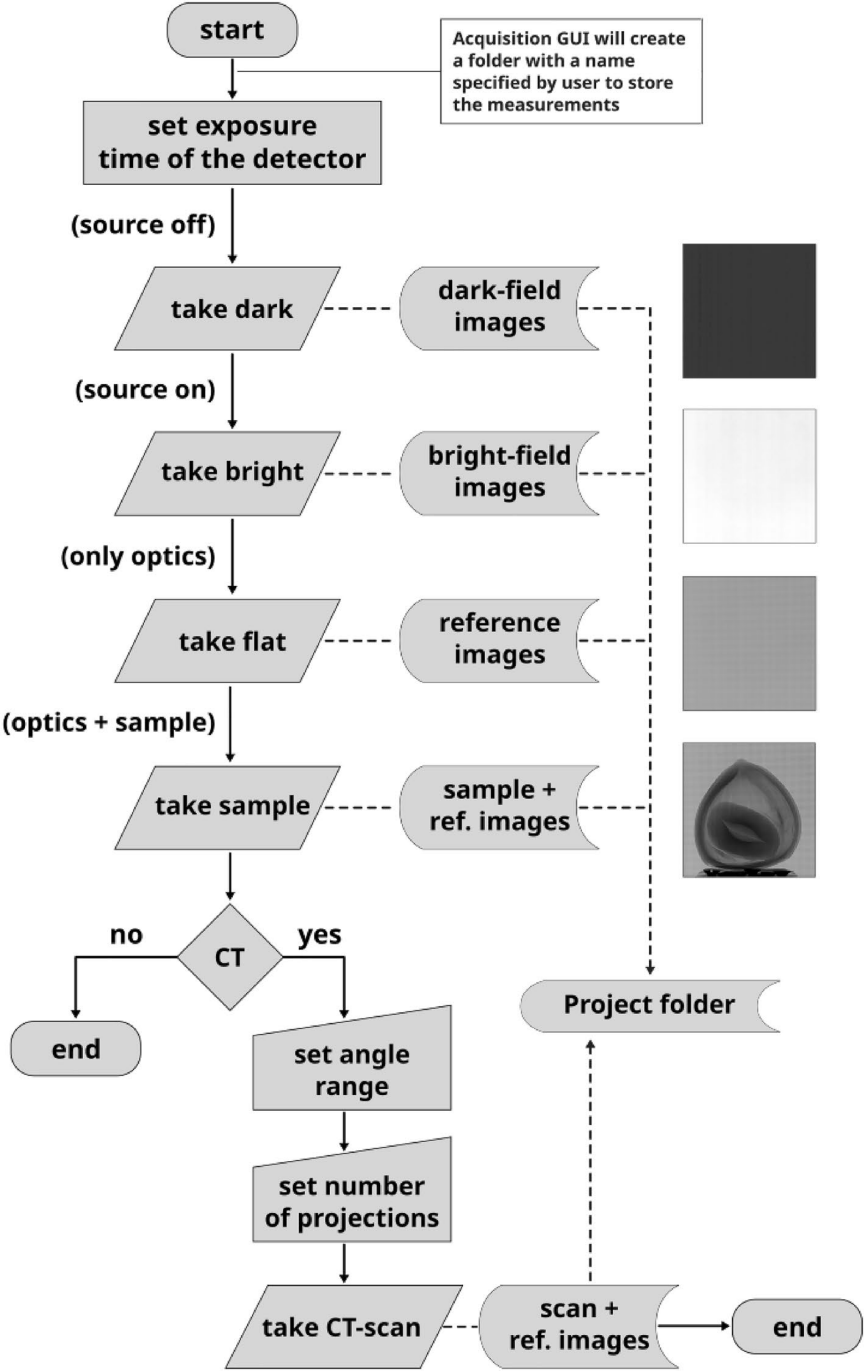
Flowchart detailing the image acquisition procedure implemented in the SHI software framework.

*SHI* automates this entire workflow for spatial harmonic imaging without compromising acquisition time, processing speed, or computational resources (such as RAM, CPU, or GPU if available). The software package is entirely implemented in Python, eliminating the need for proprietary tools and relying solely on standard modules for scientific and numerical computing (e.g., NumPy, SciPy, Matplotlib) alongside the Python standard library. It has been fully developed to operate on Ubuntu or Ubuntu-based distributions.

A key advantage of this software package is its ability to deliver high-quality results with just a few command lines. It does not require licenses or special permissions, as it is completely based on open source tools, from its libraries to the operating system, making it accessible to users with limited computational expertise.

**Fig. 11:**
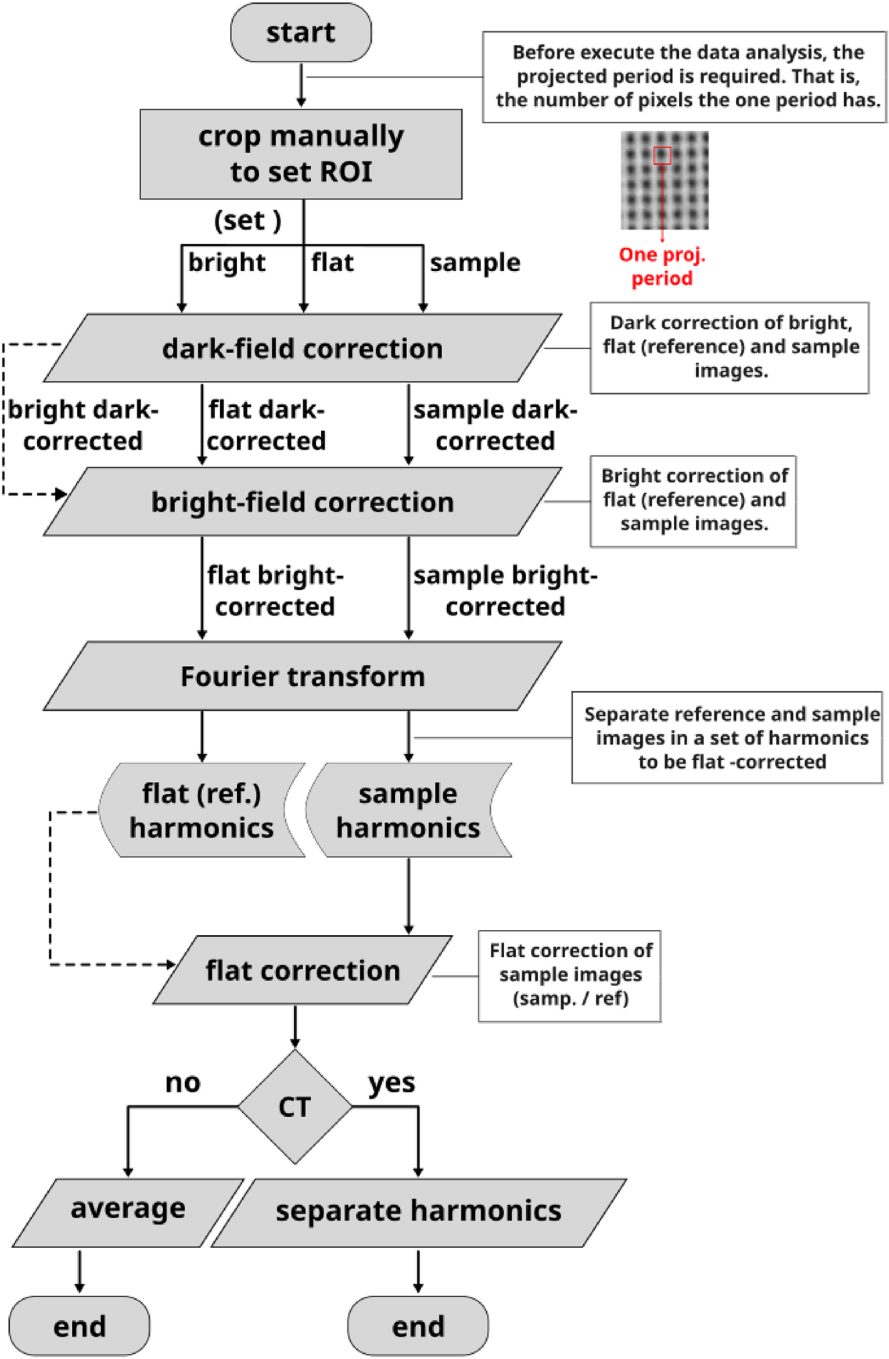
Flowchart describing the image analysis procedure implemented in the SHI software framework.

### Framework for acquisition

The experimental setup has a cone-beam geometry. The Excillum MetalJet X-ray source has the flexibility to change the spot size by software. In this case, we have set $20 \,\upmu \textrm{m} \times 80 \,\upmu \textrm{m}$ for the size of the source spot. This configuration, which uses the largest available focal spot, allows for a significant increase in the photon flux, thereby improving the SNR during acquisition. Although a larger focal spot can introduce geometric blurring owing to reduced spatial coherence, the results demonstrate that harmonic processing is robust against these effects. This experimental choice not only validates the stability of the method under less-than-ideal conditions but also represents a realistic scenario for clinical or industrial applications, where larger focal spot sizes are commonly used. The distances are 68 cm from the source to the optical component, 71 cm from the source to the sample, and 318 cm from the source to the X-ray detector. The complete Field of View provided by the detector was used so that the RAW images are $5690 \times 4608$ pixels with a bit depth of 16 bits. The pixel size of the detector is $49.5\,\upmu \textrm{m}$, thus in our configuration the effective pixel size is $11\,\upmu \textrm{m}$ and is ”over-sampled” to $52\,\upmu \textrm{m}$ due to the optical component. The exposure was fixed at 2.0 s to have a good SNR at the time of snapshots; however, it works as long as you have a signal in the detector.

**Fig. 12:**
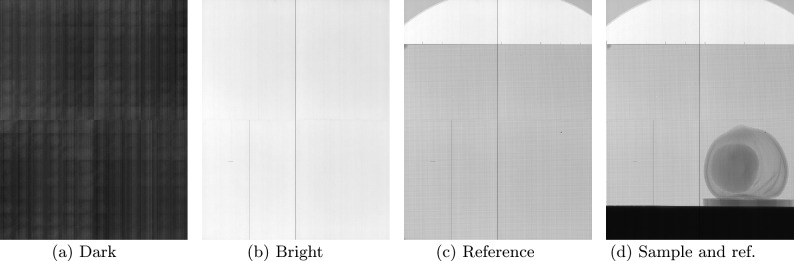
Experimental images required for the spatial harmonic imaging acquisition procedure, using a hazelnut as a test object. (**a**) Dark image: acquired with the X-ray source switched off, characterizing the detector’s intrinsic noise. (**b**) Bright image: captured with the X-ray source on and without sample or optical components, providing the flat-field illumination reference. (**c**) Reference image: recorded with the inverted Hartmann mask in place, essential for subsequent phase retrieval. (**d**) Sample image: acquired with the hazelnut positioned in the beam and the optical component maintained in the same position as in (**c**).

Before placing the sample and optical component and once the configuration is set, our software was used to acquire the dark- and bright-field images (Fig. 12a , 12b ) marked by the DARK and BRIGHT buttons (Fig. 2). The optical component is then placed and imaged by pressing the “REFERENCE” button of the GUI (Fig. 2) (resulting in Fig. 12c ). Finally, the sample can be located and imaged in two main configurations, after the optical component or before. In both cases, distances directly affect the quality of phase detection, and the sample and optical component must be radiated within the incident beam (Fig. 12d ). Although better results are obtained by acquiring several projections, contrast retrieval will also work with a single projection. In case of computed tomography, the computed tomography scan control can be executed in our GUI (see Fig. 2).

Basically, the workflow in Fig. 10 using our software will produce a project folder with all the measurements on the way for later applying the contrast retrieval process (Fig. 11). The entire acquisition workflow is shown in Fig. 10.

### Framework for contrast retrieval

Upon completion of image acquisition (Fig. 12c , 12d ), the preparation of the data is carried out as described in Fig. 11. A user-defined region of interest (ROI) is first established to constrain all subsequent computations, and thereby minimize CPU and RAM usage. ROI boundaries are saved in file and applied uniformly to the dark-field, bright-field, reference, and sample images. The dark-field correction is then applied automatically to the bright-field, reference, and sample data, followed by bright-field normalization of the reference and sample images. All corrections are performed exclusively within the cropped ROI, and the results are exported as TIFF files to a designated directory for subsequent Fourier-based processing.

**Fig. 13:**
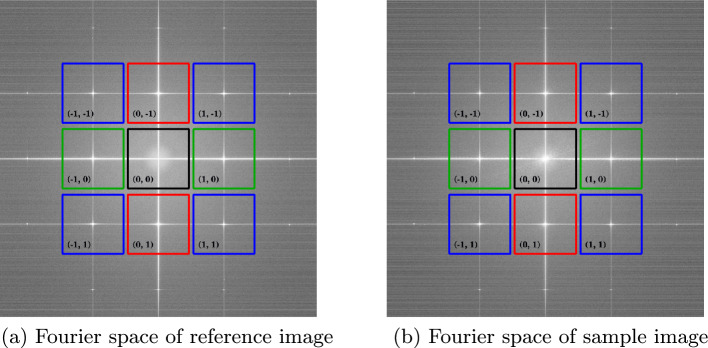
Fourier space representations of (**a**) the inverted Hartmann mask and (**b**) the sample under study with the inverted Hartmann mask placed in the beam path before the object.

Each corrected ROI undergoes a discrete Fourier transform, after which the harmonics are extracted, isolated, and assigned unique labels according to their spatial frequency. The inter-harmonic spacing depends on the projected period in pixels *d* of the grating in the detector and must satisfy $d\ge 3$ to comply with the Nyquist sampling theorem. This parameter also enables the quantification of sampling errors by comparing raw ROI data with final contrast retrieved images. To ensure consistency, the harmonics in the sample and reference Fourier spectra must occupy the corresponding quadrants (Fig. 13).

For each labeled harmonic, an inverse Fourier transform generates contrast-specific images, which are exported into separate directories according to the contrast modality (absorption, differential phase, and scattering). The final contrast maps are obtained by subtraction in pixels of sample-derived images from their reference counterparts; file names include harmonic and contrast identifiers to preserve traceability.

Exporting intermediate output serves three objectives: (1) it prevents excessive RAM consumption by offloading large arrays to the disk; (2) it facilitates stepwise quality control through visual inspection of each processing stage, allowing rapid identification of experimental misconfigurations or grating artifacts; and (3) it allows efficient averaging of multiple exposures to improve the SNR without reprocessing the entire workflow.

### Computed tomography reconstruction

In case of Computed Tomography (CT), the SHI framework also provides the same acquisition and analysis workflows by specifying the main elements for the CT scan. The difference is the organization of the directories as it is needed to separate for every projection at different angles each harmonic for further CT reconstruction.

For cone beam geometry, the well-known and classic algorithm for reconstruction is *FDK*^32^ which requires some geometric parameters. These parameters defining the geometry of the setup do not include the optical component, which in our case is the inverted Hartmann mask. However, it is possible to set an imaginary setup in which an effective pixel size is considered instead of the pixel size of the detector. In this case, the effective pixel size is calculated using the following empirical equation:

1 $$\begin{aligned} p_{\text {effective}} = p_{\text {detector}} \times \frac{D_1}{D_2}, \end{aligned}$$

where $p_{\text {detector}}$ is the detector pixel size, $D_1$ is the distance between the X-ray source and the optical component, and $D_2$ is the distance between the X-ray source and the sample. In our configuration, $p_{\text {detector}}=49.5\,\upmu$m, $D_1=68$ cm and $D_2=71$ cm, therefore, the effective pixel size is $p_{\text {effective}}=51.7\,\upmu$m.

For small source divergence angles, the acquired data can be rebinned to approximate projections^30^. After performing a CT scan from ${0}^{\circ }$ to ${180}^{\circ }$ with 2942 projections, we calculated the mean square error (MSE) between the original and rebinned data for each projection (Fig. 6). The resulting MSE values are on the order of $10^{-4}$, indicating that, using the raw or rebinned data, the subsequent reconstruction using parallel beam algorithms preserves all structurally relevant information. Consequently, only the rotation center needs to be specified as a geometric parameter when parallel-beam reconstruction routines are employed. All CT reconstructions presented in this work were performed using the *Gridrec-MS* algorithm from the *Tomopy* Python library^33^, ensuring fast, straightforward implementation and reliable structural evaluation. For applications where reconstruction fidelity is paramount, we recommend detailed geometry-aware tools such as *Astra-toolbox*^34^ or *Tofu*^35^.

## Equations

The spatial harmonic imaging technique involves an experimental configuration, as depicted in Fig. 1b , where an X-ray beam impinges on a Hartmann mask. This mask modulates the incoming wavefront and generates an array of beamlets that subsequently interact with the sample. The sample distorts those already modulated beamlets, producing various physical phenomena. The resultant intensity distribution is captured by the detector.

The intensity recorded by the detector mathematically corresponds to the convolution between the spatial frequency spectra of the object and the optical component in the Fourier space^15–17^. Given the periodic structure of the optical component, performing the Fourier transform of the recorded intensity yields a discrete set of peaks (Fig. 13), whose spatial frequency spacing is determined by the projected period of the optical component. To extract the contrasts, an inverse Fourier transform is applied individually to each harmonic component of both the reference and the sample images.

The harmonic image intensities in the real space for the reference and sample images (the inverse Fourier transform of the harmonics) can thus be expressed as follows^6,29^

2 $$\begin{aligned} I^{S, R}_{m,n} = I^{S, R}_0(x,y) D^{S, R}_{m,n}(x,y) \exp [i\psi ^{S, R}_{m,n}(x,y)], \end{aligned}$$

where *S* and *R* denote sample-plus-optic and reference images, respectively; (*m*, *n*) is the harmonic order, $I^{S, R}_0(x, y)$ represents the intensity transmitted through the optical arrangement; $D^{S, R}_{m,n}(x,y)$ corresponds to the scattering amplitude; and $\psi ^{S, R}_{m,n}(x,y)$ describes the differential phase information. However, it is necessary to eliminate the reference contribution. To extract the contrast images, we define the ratio between $I^{S}_{m,n}(x,y)$ and $I^{R}_{m,n}(x,y)$ as follows:

3 $$\begin{aligned} \frac{I^{S}_{m,n}(x,y)}{I^{R}_{m,n}(x,y)} = \frac{I^{S}_0(x,y) D^{S}_{m,n}(x,y) \exp [i\psi ^{S}_{m,n}(x,y)]}{I^{R}_0(x,y) D^{R}_{m,n}(x,y) \exp [i\psi ^{R}_{m,n}(x,y)]}. \end{aligned}$$

In Eq. (3), $I^{S}_{m,n}(x,y)$ represents the measured signal incorporating contributions from both the sample and the optical component, as recorded by the detector. This overall signal can be expressed as

4 $$\begin{aligned} I^{S}_{m,n}(x,y) = I^{S'}_{m,n}(x,y) \cdot I^{R}_{m,n}(x,y), \end{aligned}$$

which implies that

5 $$\begin{aligned} \frac{I^{S}_{m,n}(x,y)}{I^{R}_{m,n}(x,y)} = I^{S'}_{m,n}(x,y). \end{aligned}$$

In Eq. 5, the right-hand side isolates the contribution solely from the sample. It is important to note that the division in Eq. 3 is performed for the corresponding (*m*, *n*) indices (Fig. 13).

Thus, the sample-only signal is given by

6 $$\begin{aligned} I^{S'}_{m,n}(x,y) = I^{S'}_0(x,y) D^{S'}_{m,n}(x,y) \exp [i\psi ^{S'}_{m,n}(x,y)]. \end{aligned}$$

Here, $I^{S'}_0(x,y)$ denotes the transmitted intensity through the sample and is described by the Beer–Lambert law:

7 $$\begin{aligned} I^{S'}_0(x,y) = I_0(x,y) e^{-\mu (x,y) d}, \end{aligned}$$

where $I_0(x,y)$ is the intensity of the incident beam, $\mu$ is the linear absorption coefficient, and *d* is the thickness of the sample. In Eq. 6, $D^{S'}_{m,n}(x,y)$ corresponds to the scattering contrast images, and $\psi ^{S'}_{m,n}(x,y)$ yields the differential phase contrast images along the $(m,\,n)$ direction, thus facilitating the identification of preferential orientations within the sample.

Due to the absence of grating modulation in the central harmonic image $(m=0,\,n=0)$, this component is not affected by small-angle refraction or diffraction, with its attenuation resulting only from absorption and large-angle Compton scattering^17^. Therefore, for $(m=0,\,n=0)$, we have $D^{S'}_{0,0}(x,y)=1$ and $\psi ^{S'}_{0,0}(x,y)=0$, which leads to the definition of the absorption contrast image as

8 $$\begin{aligned} A_{0,0}(x,y) = -\ln \left[ \frac{I^{S}_{0,0}(x,y)}{I^{R}_{0,0}(x,y)} \right] . \end{aligned}$$

Furthermore, the harmonics $(m=0,\,n=\pm 1)$ or equivalently, $(m=\pm 1,\,n=0)$ correspond to the first-order scattering in the vertical and horizontal directions, respectively. Their scattering contrast is determined by

9 $$\begin{aligned} S_{0,\pm 1} = -\ln \left[ \frac{\left| I^{S}_{0,\pm 1}(x,y) / I^{S}_{0,0}(x,y) \right| }{\left| I^{R}_{0,\pm 1}(x,y) / I^{R}_{0,0}(x,y) \right| } \right] . \end{aligned}$$

Additionally, the differential phase contrast is obtained by computing

10 $$\begin{aligned} P_{0,\pm 1}(x,y) = \arg \left[ \frac{I^{S}_{0,\pm 1}(x,y)}{I^{R}_{0,\pm 1}(x,y)} \right] . \end{aligned}$$

For higher-order harmonics, in our case $(m=\pm 1,\,n=\pm 1)$, second-order contributions to the differential phase and scattering information can be obtained by performing

11 $$\begin{aligned} S_{\pm 1,\pm 1} = -\ln \left[ \frac{\left| I^{S}_{\pm 1,\pm 1}(x,y) / I^{S}_{0,0}(x,y) \right| }{\left| I^{R}_{\pm 1,\pm 1}(x,y) / I^{R}_{0,0}(x,y) \right| } \right] \end{aligned}$$

and,

12 $$\begin{aligned} P_{\pm 1,\pm 1}(x,y) = \arg \left[ \frac{I^{S}_{\pm 1,\pm 1}(x,y)}{I^{R}_{\pm 1,\pm 1}(x,y)} \right] . \end{aligned}$$

## Discussion

The SHI framework addresses a critical gap in spatial harmonic imaging by providing an open-source software package for the acquisition, processing, and reconstruction of multicontrast X-ray data. Its modular architecture and automation capabilities allow users, even those without specialized digital image processing expertise, to execute complex imaging workflows (Figs. 10, 11) with minimal intervention, without compromising data quality or interpretability, while remaining fully extensible for further enhancements.

The complete execution of a measurement consists of two principal stages, acquisition and processing, each of which was detailed in “Image acquisition” and “Image processing and contrast retrieval”, respectively. To date, solutions covering both stages are essentially nonexistent. Existing scripts are typically custom-built and require advanced digital image processing expertise, which undermines their reproducibility and impedes their broader application. The SHI framework addresses these issues. From a computational perspective, the SHI separates the experimental control (Fig. 10) from data processing (Fig. 11), allowing users without specialized programming skills to conduct spatial harmonic imaging experiments reliably and with optimal data quality.

In terms of acquisition, the user only needs to capture four image categories: dark, bright, reference, and sample. Accordingly, the GUI features only four buttons, each dedicated to a specific image category (Fig. 2). The output directories are automatically organized, eliminating the need to manually specify the image paths during contrast retrieval processing. Although current support is limited to Xineos GigE-Vision detectors and MCS2-SmarAct rotation stages due to resource constraints, the framework was designed for future integration of additional libraries and compatibility with other X-ray detector protocols and high-resolution stepping motors to address this limitation.

Regarding contrast retrieval, the processing stage is deliberately decoupled from acquisition, allowing independent maintenance and future enhancements without affecting other SHI components. Although the directory structures are automatically synchronized between acquisition and processing, the image paths can still be specified manually when necessary. Similar to the acquisition stage, processing was implemented entirely in Python to facilitate rapid development and cross-platform compatibility. This pure-Python design lays the foundation for a future real-time processing module, which serves as a seamless interface between acquisition and processing, thereby enabling on-the-fly contrast recovery during the live measurements. Two key features on the analysis side are the automatic extraction of harmonics (Fig. 13) and the ability to select from five phase unwrapping algorithms within a single environment. Although the latter is not the primary focus of this study, it deserves mention because of its practical significance in the field.

The workflow illustrated in Fig. 11 operates exclusively on the user-defined ROI, which explains its constant memory footprint (Table 1). However, the same calculations can be performed on full images. Our approach to harmonic extraction is entirely computational, in contrast to the original method described in earlier spatial harmonic imaging studies^15–17^. We locate each harmonic using a peak-finding routine in the Fourier domain and define a filter band limit whose spatial frequency indices tend to coincide with the midpoint between two adjacent harmonics (measured in pixels). This band limit is then propagated to all other harmonics to ensure consistent dimensionality across the spectrum.

Under ideal experimental conditions, the selected harmonic would lie exactly at the center of its Fourier space peak, and the filter band limit indices would coincide with the midpoint between the main- and first-order harmonics. However, in practice, deviations arising from the finite X-ray source size, finite detector pixel size, pixel sampling, and slight misalignment of the detector and optical component introduce small shifts. Our framework addresses these real-world imperfections by applying internal routines that estimate and compensate for residual harmonic displacements based on their expected geometric arrangement in the Fourier domain, leaving only the requirement that the optical component and detector planes are nominally parallel along their X- and Y-axes.

Due to the comparison between the harmonics of the reference image [optical component alone (see Fig. 13a)] and those of the sample image [optical component plus sample (see Fig. 13b )], harmonic extraction and labeling were performed once for a single reference image. The resulting indices and labels are then automatically applied to any additional reference or sample images. This reuse of harmonic metadata explains why the average time per image decreases as the total number of images increases (see Table 1).

**Fig. 14:**
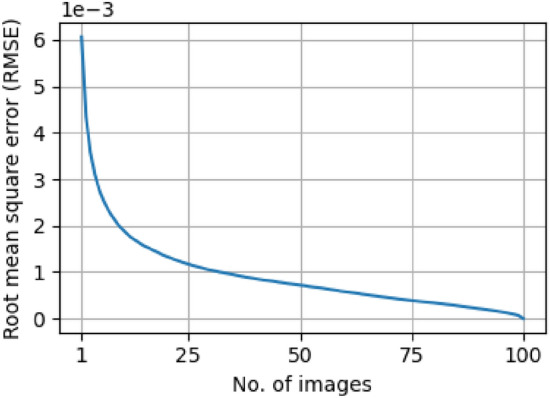
Root mean square error (RMSE) between the cumulative average of the first *n*-images and the full average over 100 images.

Regarding image quality, Fig. 6a presents a single and not averaged projection, while Fig. 3a shows a projection of the same sample obtained by averaging 100 images after contrast retrieval processing. The resulting differences are so subtle that they are barely perceptible to the eye and would only be discernible through quantitative metrics. To evaluate this effect, the RMSE between the cumulative average of the first *n* images and a reference average obtained from 100 images was used (Fig. 14). The rapid convergence of the RMSE quantifies how quickly the averaged image stabilizes as more frames are incorporated. The maximum RMSE value, corresponding to a single image, remains on the order of $10^{-3}$.

**Table 1:** Performance benchmark of the processing stage in SHI as a function of the number of input images. The table reports total execution time, average processing time per image, and peak memory usage for processing 1, 50, and 100 images.

Processing metric	1 image	25 images	50 images	75 images	100 images
Total time (s)	17.53	57.37	104.96	150.52	196.63
Average time/image (s)	17.53	2.29	2.10	2.01	1.97
Peak memory (MB)	2496.3	2495.8	2496.6	2496.1	2497.0

This approach directly influences our methodology for computed tomography (CT) reconstruction using classical parallel beam algorithms, despite the inherent cone beam geometry. To ensure a precise reconstruction, it is imperative that each projection demonstrate a high SNR. However, our objectives also include optimizing the workflow to minimize computational overhead while maintaining optimal CT reconstruction quality (Fig. 7). Ideally, a single high-SNR image per projection would suffice for homogeneous or moderately heterogeneous samples, under the acquisition and processing conditions described here. For highly heterogeneous specimens, multiple exposures may still be required to prevent photon starvation or overexposure. Although reducing to one image per angle might suggest a lower SNR, Figs. 8 and 9 demonstrate that the loss in SNR is negligible under our acquisition and processing workflows (Figs. 9, 14). Although reducing to one image per angle might suggest a lower SNR, Figs. 8 and 9 demonstrate that, under the experimental conditions and sample used in this study, the loss in SNR is negligible. These results should be understood as representative of our setup and acquisition workflow rather than a general statement applicable to all specimen types. Consequently, this strategy supports rapid low-dose CT imaging without compromising reconstruction fidelity.

Our framework does not prioritize CT reconstruction algorithms, as they have been extensively studied for both parallel and cone beam geometries. Instead, tools are provided to ensure robust preprocessing of multicontrast images prior to reconstruction. This preprocessing includes manual and automatic contrast adjustment using histogram-based methods, identification and removal of sparse or corrupted images, and correct organization of processed projection directories by the projection angle. These steps guarantee optimal input data quality for any downstream CT algorithm to be used.

To highlight the key qualitative features of SHI in its entire workflow, Table 2 provides a concise overview of its core characteristics.

**Table 2:** Benchmark of SHI key features, the spatial harmonic imaging data acquisition and processing framework.

Feature	Acquisition	Processing	CT reconstruction
Platform (OS)	Linux (Ubuntu/Debian)	Linux (Ubuntu/Debian)	Linux (Ubuntu/Debian)
Programming language	Python	Python	Python
Hardware support	Xineos GigE-V, SmarAct MCS2	N/A	N/A
User interface	GUI	CLI	GUI / CLI
Automation level	Full	Full	Full
Real-time capability	Planned	Planned	Planned
Stepwise data quality assessment	Yes	Yes	Yes
Supported file format	TIFF	TIFF	TIFF
Software license	Apache 2.0	Apache 2.0	Apache 2.0
Required programming expertise	None	None	Medium

## Conclusion

This study presents SHI, a modular software package for spatial harmonic imaging that integrates the entire workflow, including data acquisition, multicontrast image reconstruction, and CT data preparation, into a single open-source platform.

The framework implements the spatial harmonic imaging method, which is inherently robust under suboptimal conditions such as large X-ray focal spots or reduced spatial coherence. SHI preserves this robustness by implementing a computational formulation equivalent to the original analytical description of spatial harmonic imaging. In this approach, the Fourier-domain harmonic separation and contrast retrieval are performed directly from the detected frequency peaks, without assuming ideal alignment or predefined harmonic positions, yet yielding results consistent with the theoretical formulation. In our implementation, the framework maintains stable contrast retrieval and efficient performance across all tested acquisition conditions (Table 1).

The framework not only delivers robust performance under suboptimal conditions, such as a large X-ray focal spot, while maintaining stable contrast retrieval despite reduced spatial coherence, but also demonstrates excellent execution time and memory efficiency (Table 1). As the number of images increased, the average processing time per image decreased substantially without overloading the system memory. This efficiency is crucial because it makes practical real-time processing extensions of the SHI framework possible, as previously discussed. In addition, SHI establishes a foundation for the broader adoption of spatial harmonic imaging techniques and serves as a prototype for integrating additional imaging modalities within a unified software ecosystem. As the field of multicontrast X-ray imaging continues to expand, tools such as SHI will be instrumental in bridging the gap between methodological advances and practical applications.

SHI mitigates the technical complexities associated with spatial harmonic imaging, thus improving accessibility and reducing entry barriers. The benchmark data in Table 2 confirm the suitability of the SHI for laboratory integration, highlighting key features. Furthermore, the table outlines ongoing development objectives, such as adaptable hardware support and extension to other operating systems.

Spatial harmonic imaging is suitable for dynamic process investigations because of its compatibility with single-shot imaging configurations that offer a high temporal resolution.

This facilitates the integration of additional multicontrast X-ray modalities into a common, scalable software suite.

## Data Availability

The datasets generated and/or analyzed during the current study are available in the [Dataset for ”SHI: A Framework for Spatial Harmonic Imaging”] repository, https://doi.org/10.6084/m9.figshare.29617733.v1].

## Code availability

The SHI software package is available at: https://github.com/JLBeltran-IMAG/shi under the Apache License 2.0.
